# Factors associated with breast cancer screening awareness and practices of women in Addis Ababa, Ethiopia

**DOI:** 10.1186/s12905-018-0695-9

**Published:** 2019-01-07

**Authors:** S. Abeje, A. Seme, A. Tibelt

**Affiliations:** 1 Federal Ministry of Health, P.O.Box- 1234/code, 1000 Addis Ababa, Ethiopia; 2 Department of Public Health, Addis Ababa University College of Health Sciences, Addis Ababa, Ethiopia; 3 Addis Ababa University, College of Health Sciences, School of Medicine, Addis Ababa, Ethiopia

**Keywords:** Breast cancer screening, Awareness and pPractice

## Abstract

**Background:**

Breast cancer is a global health problem being the most common cancer of women in both developed and under-developed countries. Public and individual awareness can play a vital role in the prevention, early diagnosis and treatment of breast cancer. However evidence is sparse on awareness and practice of breast cancer screening methods used among women of reproductive age in Ethiopia. The present study was designed to determine factors associated with breast cancer screening awareness and practices of women in Addis Ababa.

**Methods:**

A facility based cross-sectional study was conducted among women who came for maternal and child health care services to selected public health centers. The participants were interviewed using a structured questionnaire. Following data collection, data entry and analysis was done using Epi-Info version 7and SPSS Version 21 respectively. Cross tabulation of each independent variable with the dependent variable with their 95% confidence interval was done and those variables associated at binary logistic regression with a significance level of 0.2 were entered into multiple logistic regression.

**Results:**

About half (53%) of women have heard about breast cancer and 35.5% of women are aware of at least one breast cancer screening method. Among those who are informed about breast cancer screening, 97% indicated that screening improves the chance of survival. Among the common screening methods; self-breast examination, clinical-breast examination and mammographic examination were practiced by 24.3%, 7.6% and 3.8% of respondents, respectively. Women who had high level of income were about 3 times more likely to be aware about breast cancer screening methods, [AOR = 2.5; 95%CI (1.04–-5.91)], while women who attended secondary and tertiary school were 2 and 4 times more likely to practice breast cancer screening methods [AOR = 2.46; 95% CI (1.12–-5.38)] and [AOR = 4.00; 95% CI(1.48–-10.86)] respectively.

**Conclusion:**

This study has showed that self-reported breast cancer screening coverage is low. About two-thirds of women had no information about breast cancer screening methods.

## Background

Breast cancer (BC) is a proliferation of malignant cells that arises in the breast tissue and the term represents a range of diseases from non-invasive to metastatic carcinoma [1]. It is the most common cause of cancer death worldwide for females, and the second most common cancer among all other cancers, with over 2 million new cases in 2018 [2]. It is the most commonly diagnosed cancer in women, with an age-adjusted incidence rate of 28 per 100,000 women and the second leading cause of death among women [3]. For 2018 Ethiopia, the diagnosed breast cancer incidences were15,244 (22.6%) of all cancers among men and women cancers and it accounts for 32.9%of all women cancers diagnosed. All over, the most common adult cancers were: cancers of the breast and cervix [4].Breast cancer was by far the commonest cancer and the Addis Ababa cancer registry reports that it accounts for 34% of all female cancer cases, followed by cervical cancer accounting 16% of cases [5, 6]. The disease remains a public health concern in developing and low middle income countries (DLMIC) [7, 8].

Evidence shows poor awareness of breast cancer symptoms, prevention mechanisms, risk factors and treatment options have usually been associated with patient delay in seeking help, making treatment less effective and minimal survival rate [9–11]. Screening is the most effective method to reduce mortality and morbidity from breast cancer. Screening methods like that of self-breast examination, clinical breast examination and mammography have been defined as activities facilitating the early screening and improvement of women’s health and is said to be good for early detection of breast cancer [12].

Screening is a relatively available resource for early detection though awareness about breast cancer screening is very low [13] and globally more than half the breast cancer deaths occur in low middle income countries [11]. Despite the ongoing arguing points on the benefits and harms of breast cancer screening, many experts believe that the benefits of breast cancer screening outweigh the harm [7].

There were only a limited number of studies undertaken on the awareness and practices of breast cancer screening methods among women of reproductive age in Ethiopia [14, 15]. There is scarcity of evidence on the effect of breast cancer screening in countries in which the population has limited breast cancer awareness where women typically present with late-stage breast cancer. Studies showed that when combined with appropriate treatment, early detection through breast cancer screening activities that include breast self-examination (BSE), clinical breast examination (CBE), and mammography, has been shown to decrease cancer mortality rates due to breast cancer by 25–30% [16]. This study is aimed to identify factors associated with the awareness and practices of women with regard to breast cancer screening.

## Methods

### Study setting and study design

The study was conducted in public health centers in Addis Ababa which is the largest and the capital city of Ethiopia. It covers an estimated area of 174.4 km^2^; with an estimated density of 5535.8 people per square kilometer. Based on the2007census of the Central Statistical Agency of Ethiopia, the total population estimate of Addis Ababa for the year 2015 was 3.55 million and the proportion of males and females was 49 and 51%, respectively. The number of females in the reproductive age group constitutes 35.5% of the total population. There were 84 public health centers in Addis Ababa city administration [17].A facility based cross-sectional study was conducted on women who came to maternal and child health departments of to public health centers for any kind of medical consultation, diagnosis, follow up or treatment from January to February 2015.

### Sample size, sampling procedure and ethical considerations

Data were collected from a total of 633 respondents. This sample size was based on the assumption that the population proportion with awareness of breast cancer screening would be 50% (no previous similar study), with a design effect of 1.5 due to the multistage sampling of study participants, a precision of 0.05, standardized normal distribution curve value for 95% confidence level (1.96) and a 10% non-response rate.

Recruitment of study participants was done based on sampling focused on health centers where women get maternal and child health care services. Out of the ten sub-cities four sub-cities were selected randomly. Then 10 health centers were taken from four sub-cities by a simple random sampling method where the study was conducted. The study participants were aged between 20 and 49 years and were recruited proportionally from each of the health centers using the information from patient flow, 1 month prior to the data collection. At the time of data collection all the voluntary women who came to the maternal and child health care service were interviewed consecutively according to their order of arrival until the required sample size was obtained. Data collection was conducted by experienced and trained nurses.

Ethical approval was obtained from the Institutional Review Board (IRB) of The School of Public Health (SPH) at The College of Health Sciences of Addis Ababa University (AAU).Following the approval of the IRB, Addis Ababa Health Bureau was informed about the study through a support letter from the SPH in AAU. Written permission was obtained from Addis Ababa Health Bureau to respective health facilities. Verbal informed consent was obtained from each study participant prior to data collection.

### Data collection

#### Data collection procedures

Data was collected by using a structured questionnaire after performing a thorough literature review. The questionnaire was prepared in English and then translated into Amharic. The later version was translated back to English, to ensure its consistency. There was a pre-test on 5% of the total sample size at health centers different from the selected health centers to see for the accuracy of responses, language clarity, appropriateness of the tools and the necessary amendments were done based on the findings of the pretest which were used for the actual data collection.

#### Data quality management

To maintain data quality, ten data collectors and 1 supervisor who were health professionals were selected based on their experience of data collection and were trained for 3 days. The questionnaire was developed by the principal investigator based on questions used in previous peer reviewed published articles. The collected data was reviewed and checked for mistakes, legibility of handwriting, completeness and consistency by the principal investigator and supervisor on a daily basis during data collection. Any mistake or ambiguity was cleared on the spot.

#### Data analysis procedures

The data collection instruments were coded and data were manually checked and entered using Epi-Info version 7.0. It was cleaned and edited accordingly and exported to SPSS version 21.0 for analysis. Data were rechecked for missing values before analysis.

To identify factors associated with the awareness and practices of participants of breast cancer screening, multiple questions were asked with 26 questions for assessing the general awareness of breast cancer and 6questions for assessing awareness regarding breast cancer screening. For each correct or positive answer a score of “1”was given while “0”was given for every wrong or negative answer. A mean score was calculated separately for awareness and practice variables. Any score greater or equal to the mean score was defined as high score and hence labeled as “aware” for the awareness variable. Any score which was less than the mean score was defined as a low score and hence labeled as “un aware” for the awareness. Thus, any score greater than the mean awareness of breast cancer generally was labeled as knowledgeable/aware and any score below the mean awareness was labeled as not knowledgeable or as “un aware”. Respondents who practiced any of the screening methods included in the questionnaire were labeled to have “good practice”. Respondents who never used any of the screening methods were labeled to have “poor practice”.

Descriptive statistics, numerical summary measures, frequencies, proportions, distributions were used to check for normality and also diagrams for describing the study population in relation to relevant variables. Cross tabulation and logistic regression analysis were carried out to determine the association between independent variables and the awareness and practice of breast cancer screening among the study participants with a 95% confidence interval. Those variables associated at binary logistic regression with a significance level (*p* = 0.20) were entered into multiple logistic regression to identify important determinants by controlling possible confounding effect. Statistical significance was declared at *p*-value of 0.05 and the predictors of outcome variable were identified accordingly. The reported variables were those significantly associated on adjusted odds ratio.

## Results

### Socio demographic characteristics of the study population

A total of 633 women who visit the health centers were interviewed. Majority of the respondents 422(66.7%) were orthodox Christians, 90(14.2%) Muslims, 82(12.9%) Protestants, 36(5.7%) Catholics and 3(0.5%) are others. One hundred eighty three (28.9%) of the women attended secondary school, 264(41.7%) attended primary school and 186(29.4%) attended college. Of all the respondents 482(76.1%) were married and 118(18.6%) have never been married. One hundred and sixty (25.3%) were housewives while 136(21.5%) were government employee and the rest 337(53.2) were merchants, self-employed, private employee or others.

Thirty six (5.7%) reported that they have a family history of breast illness while only 16 (2.5%) of them mentioned that they have family history of breast cancer.

### Awareness about breast cancer

From the total study participants, 336 women (53.1%) had heard about breast cancer. Among these 259 (77.1%) of them mentioned that breast cancer is non-communicable. Breast lump was mentioned by the majority (61.3%) as being a common sign and symptom of breast cancer. Smoking was recognized as a common risk factor for breast cancer and nearly 41% of the respondents mentioned initiating breast feeding and no smoking as the main preventive measures for breast cancer (Table 1).

**Table 1:** General awareness about breast cancer among women in Addis Ababa

	Mentioned variables	FREQUENCY	PERCENTAGE (%)
SIGNS AND SYMPTOMS	Breast lump	206	61.3
	Breast pain	170	50.6
	Discharge	114	33.9
	Nipple retraction	72	21.4
	Redness and engorgement	52	15.5
	Itching	16	4.8
RISK FACTORS	Smoking	150	44.6
	Alcohol	110	32.7
	Aging	71	21.1
	Being female	72	21.4
	Packed food	53	15.8
	Not to breast feed	32	9.5
	Early menarche	13	3.9
	Late menopause	16	4.8
PREVENTIVE MEASURES	Initiate Breast feeding	137	40.8
	No smoking	140	41.7
	Not drinking alcohol	124	36.9
	Regular screening	86	25.6
	Physical Exercise	72	21.4
	Combat obesity	62	18.5
	Avoid OCP	53	15.8
	Avoid packed foods	43	12.8
	Wearing bra	5	1.5

### Awareness about breast cancer screening methods

**Fig. 1:**
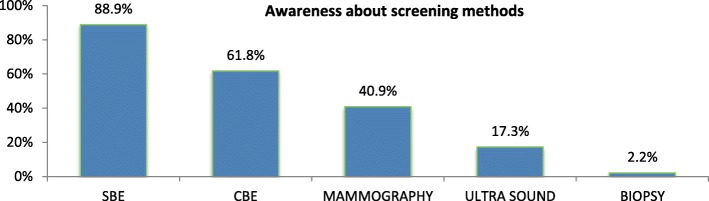
Awareness of breast cancer screening methods among women in Addis Ababa. NB: percent do not add up to 100% (multiple responses)

Among all the respondents 225(35.5%) of women are aware of at least one method of breast cancer screening. Self-breast examination was identified by 88.9% of those women who knew about breast cancer screening (Fig. 1).

### Practices of breast cancer screening methods

**Fig. 2:**
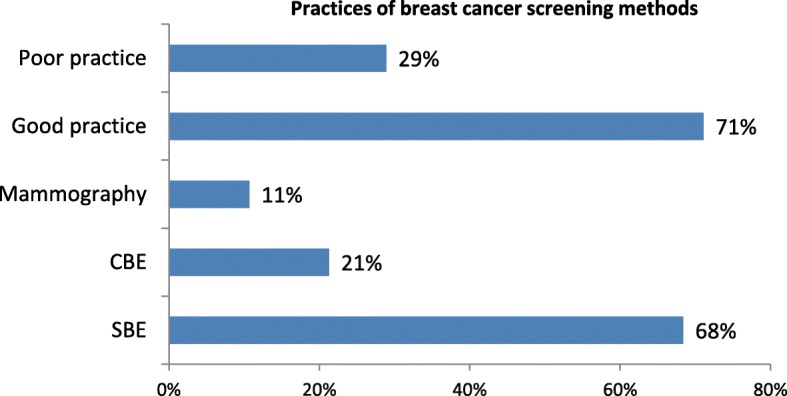
Practices of breast cancer screening methods among women in Addis Ababa. NB: percent do not add up to 100% (multiple responses)

Among those women who have heard of breast cancer screening; SBE, CBE and mammographic examination was practiced by 154 (68%), 48(21%) and 24 (11%), respectively. (Fig. 2).

Among those who are aware of breast cancer screening methods 64(28.4%) of them has reported that they performed SBE 6–12 times and 82 (36.4%) conducted 3–5 times a year. Most of them 159(70.7%) mentioned they started SBE when they were20 years or above, and 85(55.2%)reported they did SBE within the preceding month of the data collection.

Among those who have heard of breast cancer screening the commonest reason mentioned for undergoing CBE was having symptoms on breasts and wanted to check 19(39.6%) for each and having family history of BC 7(14.6%). Most 34(70.8%) of respondents have undergone CBE long time ago and only 10(20.8%) undergone CBE once a year.

Among those who were aware of BCS the commonest reason for not undergoing CBE was not having symptoms and lack of attention 153 (86%) and 19(11%) respectively.

Among those women who have heard of breast cancer screening the commonly mentioned reason for undergoing mammographic examination was they wanted to check 11(45.8%) and 7(29.2%) having symptoms.

While among those who have heard of breast cancer screening and those who do not undergo mammographic examination the commonly mentioned reason for not undergoing mammographic examination was not having symptom and not thinking to have breast cancer which counted 152(75.6%) and 24(11.9%) respectively.

### Factors associated with awareness and practice of breast cancer screening

Cross tabulation and logistic regression analysis was carried out to determine the association between independent variables and the awareness and practice of breast cancer screening among the study participants.

Educational status, occupational status, income, husband education and family history of breast illness were found to be significantly associated with the awareness of women on BCS methods on binary logistic regression while income and family history of breast illness remained significantly associated with their awareness on BCS on multivariate logistic regression.

In a multivariate logistic regression analysis those who had high level of income were about 3 times more likely to be aware about breast cancer screening compared to those with low income, **[AOR = 2.48; 95%CI (1.04–5.91)].** Those who had no family history of breast illness were 66% less likely to be aware about breast cancer screening compared to those who had family history of breast illnesses, **[AOR = 0.34; 95% CI of (0.13–0.95)]**(Table 2).

**Table 2:** Association between socio-demographic factors and the awareness of women on breast cancer screening in Addis Ababa

Variables	Mean Awareness of Screening		COR(95%CI)	AOR(95%CI)
	unaware	Aware		
Educational status				
Primary or less	54	30	1.0	
Secondary	50	52	1.87(1.04–3.38)*	1.350 (0.66–2.75)
Tertiary	36	114	5.7 (3.18–10.21)**	1.83 (0.77–4.33)
Occupation				
Merchant	14	8	1.0	
Government Employed	28	91	5.69 (2.16–14.95)**	2.58 (0.85–7.80)
Private employed	18	25	2.43 (0.84–7.01)*	1.68 (0.53–5.32)
Income				
Low income	40	47	1.0	1.0
High income	29	66	1.94 (1.06–3.55)*	2.48 (1.04–5.90)**
Husband education				
Primary education	27	12	1.0	1.0
Secondary education	50	49	2.21 (1.01–4.84)**	1.56 (0.68–3.56)
Tertiary education	38	73	4.32 (1.97–9.48)**	1.92 (0.78–4.74)
Family history of breast illness				
No	133	170	0.34 (0.15–0.82)*	0.34 (0.13–0.95)**
Yes	7	26	1.0	1.0

NB: * for p-value < 0.2 and ** for p-value < .05

On the other hand factors like educational status, religion, occupation, income, educational status of husband, occupation of husband, family history of breast illness, family history of breast cancer, and general knowledge of breast cancer were found to be significantly associated with the practice of breast cancer screening methods on binary logistic regression analysis while general knowledge of breast cancer and educational status of the women were found to be significantly associated with the practice of breast cancer screening methods on multivariate logistic regression analysis.

On multivariate logistic regression analysis those women who attended secondary school and tertiary school were 2 times and 4 times more likely to practice breast cancer screening methods compared to those who attended primary school and less **[AOR = 2.46; 95% CI (1.12–5.38)]** and **[AOR = 4.00; 95% CI(1.48–10.86)]** respectively. And those women who lack awareness of breast cancer were 99% less likely to practice breast cancer screening methods as compared to those who had good awareness of breast cancer **[AOR = 0.01; 95%CI (0.00–0.03)]**(Table 3).

**Table 3:** Association between socio-demographic factors and practices of breast cancer screening methods among women in Addis Ababa

Variables	Practices of screening methods		COR (95%CI)	AOR(95%CI)
	Those who did not practice	Those who practiced		
Educational status				
Primary and less	230	34	1.0	1.0
Secondary	118	65	3.73 (2.33–5.97)**	2.46 (1.12–5.38)**
Tertiary	60	126	14.21 (8.85–22.81)**	4.00 (1.48–10.86)**
Income				
Low income	159	55	1.0	1.0
Middle income	177	97	1.58 (1.07, 2.35)**	0.93 (0.39–2.21)
High income	72	73	2.93 (1.87–4.58)**	1.99 (0.75–5.29)
occupation				
Merchant	82	20	1.0	1.0
Government employed	53	50	3.90 (2.07–7.21)**	2.21 (0.70–6.96)
Private employed	84	46	2.25 (1.22–4.12)**	2.08 (0.69–6.29)
Self employed	81	37	1.87(1.00–3.50)**	1.48 (0.49–4.41)
Husband education				
Primary education	136	14	1.0	1.0
Secondary education	115	59	4.98 (2.65–9.39)**	1.64 (0.66–4.07)
Tertiary education	74	84	11.03 (5.86–20.76)**	1.50 (0.54–4.14)
Family history of breast illness				
No	402	195	0.10 (0.04–0.24)**	0.24 (0.05–1.15)
Yes	6	30	1.0	1.0
Knowledge of breast cancer				
Not knowledgeable	322	7	0.01 (0.00–0.02)**	0.01 (0.00–0.03)**
Knowledgeable	86	218	1.0	1.0

NB: * for p-value < 0.2 and ** for p-value < .05

## Discussion

This study assessed the factors associated with breast cancer screening awareness and practices of women. The study also described the awareness of breast cancer, demographic characteristics that affect breast cancer screening. It was identified that factors such as family history of breast cancer, income level associated with the awareness of breast cancer screening.

A high level of awareness about breast cancer screening is important for success of prevention intervention. Evidence shows awareness of breast cancer screening varied from 10 to 80% [18], this study showed 89% of participants know about SBE.

Our study showed 53.1% of the women are aware of breast cancer. A study conducted in an urban area of India showed that 56% of women were aware of breast cancer; while a study conducted in Iran showed 61% of women knew about breast cancer [13, 19]. In all studies more than half of the study participants knew about breast cancer, which might indicate that there was a slightly higher health seeking behavior for breast cancer screening in study participants.

This study showed that over half of the study participants had an awareness score of greater than or equal to the mean awareness score for breast cancer symptoms such as: breast lump, breast pain and discharge as a common symptom for breast cancer. This was higher than in a study conducted in Northern Ethiopia [15]. The difference could be attributed to the urban nature of this study area since they would have better access to information on breast cancer.

This study revealed that 31.6% of participants were able to correctly identify breast self-examination (BSE) but was less compared to a study from Nigeria. This could be due to the socio-demographic differences between the two study areas. What was striking in this study was that 14.5% of women (knew) mammography as a method for breast cancer screening and which was higher than the study done in Ethiopia and Nigeria [15, 20], this could be due to the time of the study. There was better awareness in the current study period compared to the previous studies.

Our study showed that factors like family history of breast cancer and income is associated with the awareness of women about breast cancer, in which family history of breast illness perhaps gave them a chance to have an information and increases health seeking behavior. When a family has better income it will also increase access to education and information. These findings were contrary to study conducted in Northern Ethiopia [15], but consistent with community based study in Tunisia [21]. In other studies education, cultural factors, psychosocial and socio-demographic factors were identified as barriers for breast cancer screening [15, 22]. Successful breast cancer screening program in low-middle income countries needs the identification of potential barriers and the development of effective strategies to address them.

SBE, CBE and mammographic examinations were practiced by 24.3, 7.6 and 3.8%, respectively. This is lower than the study done in Nigeria. And this could be due to the differences in socio-economic factors between the study areas. And the main reason mentioned for not having clinical breast examination (CBE) was not having symptoms 86.4% which was similar to the study done in Benin Nigeria [22].

Majority of the participants in this study (86.4%) mentioned absence of symptoms as the main reason for not undergoing CBE and this is similar to the study of north Ethiopia [15]. The similarity could be due to the health seeking behavior of the two study areas were similar and this showed women do not practice unless they have symptoms.

This study showed only (3.8%) had mammographic examination slightly higher than the study of North Ethiopia; while the main reasons for non-practice of mammographic examination were not having symptoms, lack of attention to BC, and its expense 75.6, 11.9 and 6.5% respectively. While in the study of north Ethiopia the main reasons given by the women for not having mammographic examination were, doubt about its importance 33.8%, lack of awareness about it is 31.9% and unavailability of the service 14.0% [15]. And this difference could be due to the relatively better availability of mammography in urban city. In this study it was found that those who had higher income (> 1500 Ethiopian birr) were 3 times more likely to be aware about breast cancer screening methods as compared to those who had low income. Logically, being knowledgeable about the methods has to lead the respondents to practice the screening methods, the binary regression showed there is an association but the adjusted odds ratio was not significant and we believe only being aware about the methods is not enough to initiate the practice and there also might be other confounding factors.

A limitation of the study is the cross-sectional nature of the data made it impossible to reach at the causal relationship between the different independent and outcome variables. Lack of literatures from developing countries hinders further discussion and comparison.

## Conclusion

This study highlights the need to address the awareness and practice of breast cancer screening among women. Differences in awareness related outcomes analyzed according to socio-demographic and other factors noted in the results suggest potential target groups for future. These suggest that local and national activity might work in collaboration to address a wide range of challenges identified in this study.

## Abbreviations

AAU: Addis Ababa University
AOR: Adjusted Odds Ratio
BC: Breast Cancer
BCS: Breast Cancer Screening
BSE: Breast Self-Examination
CBE: Clinical Breast Examination
CI: Confidence interval
IRB: Institutional Review Board
